# Effect of Thyme (*Thymus vulgaris* L.) Used in Diets with Extruded Flaxseed on the Antioxidant and Lipid Profile of the Blood and Tissues of Fattening Pigs

**DOI:** 10.3390/antiox12051045

**Published:** 2023-05-04

**Authors:** Kamila Klimiuk, Iwona Sembratowicz, Krzysztof Tutaj, Anna Czech

**Affiliations:** Department of Biochemistry and Toxicology, Faculty of Animal Sciences and Bioeconomy, University of Life Sciences in Lublin, Akademicka 13, 20-950 Lublin, Poland

**Keywords:** thyme additive, flaxseed, pig tissue, antioxidant parameters, fatty acids

## Abstract

Thyme has strong antioxidant properties and, therefore, can reduce the intensity of oxidative processes taking place in the body. The study aimed to assess whether the addition of thyme to diets for fattening pigs containing extruded flaxseeds, a source of n-3 PUFAs, which are particularly susceptible to oxidation, would have a positive effect on redox status and lipid metabolism. The experiment was conducted using 120 weaners (WBP × Neckar crosses) of about 30 kg BW, which were kept until the end of fattening (about 110 kg BW) and divided into three groups of 40 pigs. The control group received a diet with 4% extruded flaxseed. In groups T1 and T3, 1% or 3% of thyme was added to the basal diet. The introduction of 3% thyme resulted in a decrease in the total cholesterol level in the blood and the loin muscle. Moreover, an increase in SOD and CAT activity and a decrease in FRAP and LOOH was noted. Following supplementation with 3% thyme, the n-3 PUFA content and n-3/n-6 ratio increased, while the SFA content was significantly reduced. The results of the studies indicate that thyme has a positive effect on the redox status and lipid profile of the blood and muscles.

## 1. Introduction

Due to health concerns, consumers are increasingly paying attention not only to the nutritional value of food products but to their health benefits as well. For this reason, components are added to diets for livestock in order to obtain products with health-promoting properties, e.g., with high content of polyunsaturated fatty acids (PUFA), especially n-3 acids. One ingredient used for this purpose is flaxseed, which improves meat quality due to increased content of PUFAs, such as α-linolenic (ALA), eicosatrienoic, eicosapentaenoic acid (EPA), and docosapentaenoic (DPA) acids [1]. This is a positive phenomenon, but PUFAs are highly susceptible to oxidation, which leads to redox imbalances in the body and reduces the nutritional value and quality of food [2]. This process leads not only to the degradation of valuable unsaturated fatty acids but also to losses of some vitamins [3] and reduced utilization of protein from feed [4]. Furthermore, lipid oxidation results in the formation of harmful metabolites for consumers, such as aldehydes, ketones, or peroxides [5]. Therefore, antioxidants such as tocopherols or selenium must be added in order to protect fat against oxidation. Although flaxseed contains substances with antioxidant properties (e.g., lignans and vitamin E), their quantity may not appear sufficient to effectively limit oxidation processes resulting from the presence of polyunsaturated fatty acids. An effective solution applied in practice is to supplement animal diets with plant preparations known as phytobiotics. These include herbs with antioxidant properties, rich in phenols and other antioxidants. Martini et al. [6] analysed the effect of the combined use of polyphenol-rich extracts of red grape skin, oregano, and extruded flaxseed on oxidation processes in raw, grilled, and in vitro-digested pork. The addition of natural antioxidants was shown to reduce oxidation processes during cooking and digestion and to be more effective than the use of synthetic vitamin E and sodium selenite. A significant observation was that polyphenols are accumulated in the muscle tissue, which most likely determines their beneficial effects in it.

Herbs of the family Labiatae are particularly rich in antioxidants. These include many medicinal and seasoning herbs, such as rosemary, thyme, oregano, sage, and mint [7]. Among herbs of the family Labiatae, thyme is worthy of attention. Extracts of thyme exhibit particularly high antioxidant activity [8] due to its rich and highly diverse chemical composition. The antioxidant properties of thyme are exhibited not only by oil components such as thymol and carvacrol but by other phenolic substances as well [9]. By reducing the oxidative degradation of PUFAs, phytobiotics (e.g., thymol and carvacrol) help to increase the content of these valuable acids in the muscles [10].

Phenolic compounds and other active plant substances can have beneficial effects on numerous biochemical processes in the body, including lipid metabolism, which leads to a decrease in the level of cholesterol and its atherogenic LDL fraction. Herbs with this type of activity include basil, ginseng, dandelion, and thyme [11,12]. The possibility of including natural antioxidant sources such as thyme in the diet of pigs in order to improve their health condition and increase the nutritional and health-promoting value of their meat is very interesting, especially as no research has yet been conducted on the effect of thyme herb on the redox and lipid profile of the blood and tissues of fattening pigs.

The aim of the study was to determine the effect of the administration of compound feed with 4% inclusion of extruded flaxseed in combination with different levels of thyme herb (1% or 3%) on lipid parameters and redox processes in fattening pigs, as well as on the oxidative stability and fatty acid profile of the tissues.

## 2. Materials and Methods

The material for the research was WBP × Neckar crossbred weaners, from a body weight of about 30.32 ± 0.24 kg (about 84 days of age) until the end of the fattening period (about 112.2 ± 3.79 kg BW; 168 days of age). The animals were individually tagged, weighed, and assigned to three feeding groups matched for sex and body weight. Each group comprised 40 animals, kept in 5 pens with 8 pigs in each (4 gilts and 4 barrows). The animals received complete Grower and Finisher diets ad libitum. The content of nutrients, amino acids, phosphorus, and calcium in all groups met the nutritional requirements laid out in NCR for pigs [13] (Table 1).

All experimental procedures used in the study were approved by the Second Local Ethics Committee on Animal Experimentation of the University of Life Sciences in Lublin, Poland.

The pigs in the control group received a diet containing extruded flaxseed in the amount of 4%. This composition was chosen on the basis of previous research [14,16], which showed the beneficial effects of extruded flaxseed on the lipid profile and redox processes in pigs, as well as on the oxidative stability and fatty acid profile of raw pork. Despite these benefits, to additionally enhance antioxidant defense and protect PUFAs against oxidation, as well as to improve gastrointestinal function, thyme was added to the basal diet in the amount of 1% (group T1) or 3% (group T3).

The choice of thyme was influenced by a pilot experiment involving observations of the food preferences of piglets, which were given diets containing herbs with antioxidant properties: thyme, caraway, coriander, and purple coneflower. The observations showed that the diet containing thyme was most preferred by the piglets. Dried thyme herb was purchased from the herb shop Zakład Zielarski KAWON-HURT Nowak (Gostyń, Poland) and added to the diets when they were being prepared in the feed mixer.

### 2.1. Experimental Procedures

The feed was sampled three times during the experiment and analysed for the content of basic nutrients (crude protein, crude fibre, crude fat, lysine, methionine, and cysteine) and minerals (phosphorus and calcium) according to AOAC [17].

The thyme herb was analysed as well.

The samples were homogenized using a BUCHI mixer B-400 with ceramic blades.

The homogenates were analysed for total phenolic content, total antiradical activity using DPPH and ABTS radicals, and flavonoid content.

Thyme was extracted by a modification of the method described by Bakowska-Barczak and Kolodziejczyk [18]. Dry thyme (0.1 g) was extracted twice with 1 mL of 80% aqueous methanol containing 0.1% formic acid for analysis of total phenolic content and evaluation of antioxidant capacity. Total phenolic content (TPC) was determined according to Song et al. [19].

The ABTS•+ assay was carried out according to Re et al. [20]. DPPH• free radical scavenging activity was measured according to Bocco et al. [21]. Total flavonoid content was measured according to Dewanto et al. [22]. The content of individual substances was determined according to Czech et al. [23].

#### 2.1.1. Analysis of Animal Material

Blood was sampled for analysis from 6 barrows from each group at a body weight of about 70 and 100 kg. The animals had no access to feed for 12 h before blood sampling. Blood was drawn from the jugular vein into 10 mL heparinized tubes. The pigs chosen for analysis were matched for body weight. The same animals from which blood was sampled were slaughtered at about 110 kg BW, and the material collected from them was used for further analysis.

#### 2.1.2. Blood Analysis

Plasma was obtained by centrifuging whole blood at 3500 rpm for 15 min in a laboratory centrifuge (MPW-260R, Warsaw, Poland) and stored in Eppendorf tubes at −80 °C until analysis.

#### 2.1.3. Biochemical Parameters

Tests from Cormay (Lublin, Poland) were used to analyse the plasma for selected biochemical parameters, i.e., total protein (TP; Cor-TOTAL PROTEIN 60, catalogue No. 2-236, Poland), uric acid (UA; Liquick Cor-URIC ACID 120, catalogue No. 2-208, Poland), urea (UREA; Liquick Cor-UREA 60, catalogue No. 2-206, Poland), creatinine (CREAT; Liquick Cor- CREATININE 60, catalogue No. 2-233, Poland), phosphorus (P; Liquick Cor-PHOSPHORUS 120, catalogue No. 2-338, Poland); total cholesterol (CHOL; Liquick Cor-CHOL 60, catalogue No. 2-204, Poland), triacylglycerols (TG; Liquick Cor-TG 30, catalogue No. 2-262, Poland), and HDL cholesterol (HDL-C; Liquick Cor-HDL, catalogue No. 2-053, Poland). LDL cholesterol (LDL-C) was calculated using the formula given by Friedewald [24]:

LDL-C (mmol/l) = CHOL–HDL-C-TG/2.2

Tests from Cormay (Lublin, Poland) were used to determine the activity of alkaline phosphatase (ALP; Liquick Cor-ALP 60, catalogue No. 1-212, Poland), alanine aminotransferase (ALT; Liquick Cor-ALAT 60, catalogue No. 1-216, Poland), and aspartate aminotransferase (AST; Liquick Cor-ASAT 60, catalogue No. 1-214, Poland).

Ca, Mg, Zn, Cu, and Fe content was determined by atomic absorption spectrometry (AAS) with a Varian model 720-ES ICP-OES spectrophotometer (Varian, Palo Alto, Santa Clara, CA, USA).

#### 2.1.4. Redox Parameters

The activity of antioxidant enzymes superoxide dismutase (SOD) and catalase (CAT) and the concentrations of lipid peroxidation products, i.e., peroxides (LOOH) and malondialdehyde (MDA), were determined in the plasma and tissues and parameters of the antioxidant system were measured in the plasma, i.e., ferric reducing antioxidant power (FRAP) and content of vitamin C, according to methods described by Czech et al. [25].

The contents of fatty acids and cholesterol were analysed following fat extraction with a chloroform/methanol mixture, according to Folch et al. [26]. The analysis was conducted according to standards [27,28].

The percentages of fatty acid methyl esters were estimated by gas chromatography on a Varian CP-3800 chromatograph. The operating conditions for fatty acid separation were as follows: CP WAX 52CB DF 0.25 mm capillary column 60 m in length, gas carrier–helium, flow rate 1.4 mL/min, column temperature 120 °C gradually increased by 2 °C/min to 210 °C, determination time 127 min, feeder temperature 160 °C, detector temperature 160 °C, other gases—hydrogen and oxygen.

The content of fatty acids in the longissimus dorsi muscle was calculated according to Weihrauch et al. [29]. Lipid quality indicators, i.e., the atherogenicity index (AI) and thrombogenicity index (TI), were calculated using the following equations [30]:

AI = [(4 × C14:0) + C16:0]/[n-6 PUFA + n-3 PUFA + MUFA]

TI = [C14:0 + C16:0 + C18:0]/[(0.5 × MUFA) + (0.5 × n-6PUFA) + (3 × n-3PUFA) + n-3/n-6 PUFA]

### 2.2. Statistical Analysis

All data are expressed as means and SEM (standard error of the mean).

Data were analysed by ANOVA with treatment as the fixed effect and pig (tissues and blood analysis) as the experimental unit.

The normality of the data distribution was tested using the Shapiro–Wilk test and equality of variance was tested by Levene’s test.

Treatment means were compared using Tukey’s HSD (honest significant difference) test in Statistica 13 [31].

For all tests, a criterion α level of *p* < 0.05 was used to determine statistical significance.

## 3. Results

The content of chemical components, including the antioxidant parameters of thyme herb, is presented in Table 2.

The 3% inclusion of thyme in the diets in the grower period caused a significant decrease in the total cholesterol level compared to the control group (*p* = 0.003) and in the finisher period compared to the control group and T1 group (*p* = 0.001). The plasma of fatteners in the grower period receiving a diet with 1% thyme had a significantly lower level of TG than in the control (*p* = 0.015). In the grower period, the plasma of pigs receiving a diet with 1% and 3% thyme had a significantly higher content of HDL-C than in the control (*p* = 0.046). In the finisher period, the HDL-C content in the plasma of pigs receiving a diet with 3% thyme (T3) was significantly higher than in the control (*p* = 0.012). The plasma of group T3 fatteners had a significantly lower content of LDL-C (*p* = 0.044 grower and *p* = 0.003 finisher) compared to the other groups. The level of thyme significantly influenced the content of CHOL (*p* = 0.006 finisher), HDL-C (*p* = 0.013 finisher), TG (*p* = 0.013 finisher), and LDL-C (*p* = 0.016 grower and *p* = 0.005 finisher) (Table 3).

The levels of uric acid and urea in the plasma of finisher pigs receiving a diet with 3% inclusion of thyme were significantly higher than in the other experimental groups (*p* = 0.044 and *p* = 0.009, respectively). The level of inclusion of thyme also significantly influenced UA and UREA in the plasma of finisher pigs (*p* = 0.048 and *p* = 0.11, respectively) (Table 4). Alkaline phosphatase activity in the plasma was significantly increased by the addition of thyme (*p* < 0.001 grower and *p* = 0.005 finisher) relative to the control and was highest in the group receiving a diet with 3% thyme (*p* = 0.039 grower and *p* = 0.009 finisher) (Table 4).

Superoxide dismutase activity and vitamin C content were significantly higher in the plasma of pigs in group T3 than in groups C and T1 (grower period: SOD-*p* < 0.001, Vitamin C-*p* = 0.001; finisher period: SOD–*p* = 0.047, Vitamin C-*p* = 0.033). Ferric-reducing antioxidant power (FRAP) in the plasma of grower pigs from groups C and T3 was higher than in group T1 (*p* = 0.001), while in the finisher period, the FRAP value was significantly higher in the plasma of pigs receiving a diet with thyme (T1 and T3) than in the control (*p* = 0.004). LOOH content in the plasma of finisher pigs (*p* = 0.020) and MDA in grower (*p* = 0.001) and finisher (*p* = 0.009) pigs were significantly lower than in the control (Table 5).

SOD and CAT activity was significantly higher in the longissimus dorsi muscle of pigs from the group receiving a diet with 3% thyme compared to the control (*p* = 0.016 and *p* = 0.039, respectively). The level of inclusion of thyme significantly influenced CAT activity (*p* = 0.026). The content of LOOH and MDA in the longissimus dorsi muscle of pigs from group T3 was significantly lower than in the control (*p* < 0.001 and *p* = 0.038, respectively). The level of thyme significantly influenced the content of both LOOH (*p* = 0.041) and MDA (*p* = 0.049) (Table 6).

In the case of fatty acids, the inclusion of thyme in diets for fattening pigs significantly reduced the concentration of 14:0 (*p* = 0.016) in the longissimus dorsi muscle relative to the control. The longissimus dorsi muscle of group T3 fatteners had significantly lower content of 18:0 (*p* = 0.050) and higher content of 20:1 (*p* = 0.049), 18:2 (*p* = 0.023), 18:3 (*p* = 0.039), and 20:3 (*p* = 0.045) compared to the other groups. The level of thyme significantly influenced the levels of 18:0 (T1 > T3, *p* = 0.046), 20:1 (T1 < T3, *p* = 0.011), 18:2 (T1 < T3, *p* = 0.022), 18:3 (T1 < T3, *p* = 0.048), and 20:3 (T1 > T3, *p* = 0.022) (Table 7).

The content of SFAs in the longissimus dorsi muscle of pigs from group T3 was significantly lower than in the other experimental groups (*p* = 0.021), while the PUFA content in the longissimus dorsi muscle of pigs from group T3 was significantly higher than in group C (*p* = 0.029). Significantly higher content of n-3 acids (*p* = 0.028) and a higher n-3/n-6 ratio (*p* = 0.046) were noted in the muscle of T3 pigs compared to the other experimental groups (C and T1 groups). The 3% inclusion of thyme in diets for pigs caused a significant reduction in the TI index (*p* = 0.043) and the cholesterol level (*p* = 0.013) compared to the control. The level of thyme inclusion significantly influenced the content of SFAs (T1 > T3, *p* = 0.041), the content of n-3 acids (T1 < T3, *p* = 0.027), and the n-3/n-6 ratio (T1 < T3, *p* = 0.037) (Table 7).

## 4. Discussion

Formulation of diets for pigs presents many challenges associated with the choice of feed ingredients that will provide adequate amounts of essential nutrients and, at the same time, ensure that the animal products will meet consumer expectations. Consumers prefer products with the highest possible levels of substances with health-promoting effects, such as valuable protein, minerals, and polyunsaturated fatty acids (PUFAs). A valuable source of PUFAs for animals, especially n-3 PUFAs, is flaxseed, which is increasingly used as a feed ingredient for various livestock animals. This strategy results in beneficial modifications of the fatty acid composition of the meat or other products, such as milk or eggs. Our earlier research [16] and studies by other researchers [32] have shown that the inclusion of flaxseed in the diet of pigs increases the content of beneficial omega-3 fatty acids in animal tissues and also has a positive effect on the lipid profile of the blood. Due to the high susceptibility of PUFAs to oxidation, whose rate increases with the number of unsaturated bonds in the molecule [33], the use of antioxidant additives is recommended. Popular additives that can minimize lipid oxidation and positively affect the lipid profile of the blood include various phytobiotics, including herbs and their extracts, such as oregano (100, 200 mg/kg) or thyme essential oils (300, 450 mg/kg) [34,35].

In the present study, the diet containing thyme herb in the amount of 3% had a beneficial effect on the lipid metabolism of fattening pigs, resulting in a reduction in the level of cholesterol and its LDL fraction, as well as that of TG even in the case of 1% inclusion of the phytobiotic. Shao et al. [36] used a blend containing carvacrol, thymol, and cinnamaldehyde in the diet of early-weaned piglets and found that it caused a decrease in TG, total cholesterol, and its LDL-C and HDL-C fractions. A reduction in the concentrations of total cholesterol and triacylglycerols was also noted in calves receiving a diet supplemented with thyme or oregano essential oils [37]. Similar results have been reported in poultry, e.g., quail [38] and chicken broilers [39].

Lipid metabolism is regulated mainly by the active substances in thyme oil, i.e., thymol and carvacrol. These substances have been shown to exert an inhibitory effect on the activity of hydroxymethylglutaryl–coenzyme A reductase, a key enzyme regulating endogenous biosynthesis of cholesterol [40]. The capacity of the active components of thyme to reduce the TG level in the blood may be linked to the stimulation of lipoprotein lipase activity, as shown in research using other herbs [41].

There are indications that thyme has hepatoprotective effects in the case of damage to the liver caused by various factors [42]. The beneficial effect of thyme on liver function was manifested as a reduction in the activity of indicator enzymes AST and ALT. However, long-term intake of high doses of formulations containing essential oil components thymol and carvacrol has been shown to have the opposite hepatotoxic effect [43]. Therefore, caution and monitoring of liver markers are recommended during the administration of thymol or carvacrol. Analysis of the effect of the inclusion of thyme in the diet of fattening pigs in the present study did not reveal changes in the values of these liver indicators (ALT or AST) or of kidney markers (CREAT, UREA, UA), which suggests that this level of the phytobiotic does not disturb liver or kidney function. Improvement of kidney and liver function in rabbits fed diets with aqueous extracts of thyme (50 mg/kg BW) was reported by Abdel-Gabbar et al. [44]. The researchers noted a decrease in kidney markers, i.e., creatinine, uric acid, and urea, as well as in the activity of ALT and AST. These effects are mainly attributed to the antioxidant properties of thyme, which, like other plants of the family Labiatae, is a rich source of phenolic substances: phenolic acids (primarily rosmarinic acid) and flavonoids. Apart from their effect on redox status, the active compounds in thyme may have anti-inflammatory properties and also increase the activity of phase I and II xenobiotic detoxification enzymes, which also favours hepatoprotection [45].

The results of the present study, in which the effect of thyme herb on redox parameters of the blood was evaluated, confirmed the antioxidant effects of the plant. The plasma of pigs receiving a diet with 3% thyme herb showed increased activity of antioxidant enzymes SOD and CAT, which protect tissues against oxidation, as well as an increased FRAP value (ferric reducing antioxidant power). The value of this parameter is determined by the presence of vitamin C (15%), uric acid (60%), bilirubin (5%), and protein (10%) [46]. The level of low-molecular-weight antioxidants (uric acid, urea, and bilirubin) in the blood of pigs receiving a diet with 3% thyme was higher than in the control group but was still within reference values [47]. The content of vitamin C was also significantly higher in the group receiving 3% thyme. A study using rats with hyperlipidaemia [11] showed that polyphenol-rich extracts of various varieties of thyme exhibit significant antioxidant activity, as indicated by FRAP and RSA (Radical Scavenging Activity) values, owing to which they can protect organs from oxidative stress. Polyphenols have been shown to exhibit multi-faceted antioxidant activity, including scavenging of free radicals, inhibition of enzymes taking part in free radical production, and chelation of metal ions [48]. Furthermore, these compounds can protect natural antioxidants such as tocopherols or vitamin C against oxidation [49]. A study by Placha et al. [50] using rabbits confirmed that the addition of exogenous antioxidants, such as antioxidant-rich thyme oil, can improve the body’s redox status, increasing the total antioxidant status of the plasma and GPx activity in the liver. In the present study, enhancement of endogenous antioxidant defense in pigs receiving a diet supplemented with thyme herb resulted in a reduction in indicators of lipid peroxidation in the blood, i.e., LOOH and MDA. The results pertaining to the levels of antioxidants and activity of antioxidant enzymes in the muscle tissue of the pigs indicate similar tendencies to those noted in the blood. The content of MDA and LOOH at both levels of thyme supplementation (1% and 3%) was significantly reduced, with a much more pronounced decrease in the case of 3% inclusion. Therefore, the higher inclusion of thyme inhibited lipid oxidation to a greater extent and also caused a significant increase in the activity of enzymes involved in antioxidant processes, i.e., SOD and CAT. Martini et al. [6] added natural extracts of grape skin and oregano to the diet of pigs receiving feed with extruded flaxseed. This resulted in a reduction in the amount of lipid oxidation products (hydroperoxides and TBA-RS) in the grilled pork, as well as in meat digested in vitro. In the group of pigs receiving synthetic antioxidants, this effect was observed only in the cooked meat but not in the digested meat. Studies by various authors indicate that supplementation of animal diets with antioxidant substances such as thymol, carvacrol, or oregano oil can have a beneficial impact by reducing oxidation phenomena in the meat [51,52,53]. In the case of poultry, a reduction in lipid oxidation products during meat storage was obtained by using thymol or carvacrol as feed additives (150 mg/kg), which proved to be equally as effective as BHT [51]. Ranucci et al. [52] used a combination of oregano essential oil and sweet chestnut wood extract (0.2%) to supplement feed for pigs and observed a beneficial increase in glutathione peroxidase and glutathione reductase activity in the longissimus lumborum muscle, accompanied by a decrease in lipid oxidation indicators. A trend of decreasing markers of lipid oxidation in pork following feed supplementation with oregano oleoresin (0.05%) was reported by Janz et al. [53].

The inclusion of thyme herb in the diet of pigs also modified the fatty acid profile of the loin meat. Both levels of the additive reduced the content of acids 14:0 and 18:0 and the total amount of saturated fatty acids. The use of 3% thyme herb increased the content of acids 20:1, 18:2, 18:3, and 20:3, the total PUFA content, and the n-3/n-6 ratio. In addition, there was a decrease in the thrombogenicity index TI and the cholesterol content. These changes should be regarded as highly favourable for consumers of pork, as a lower intake of saturated fatty acids and cholesterol can minimize the risk of atherosclerosis and other cardiovascular diseases [54]. The inclusion of thyme in the diet (group T3) inhibited the oxidation of unsaturated fatty acids, especially PUFAs, which are more susceptible to oxidation in the loin muscle of pigs. This resulted in an increase in the content of PUFAs in the muscle tissue.

This is also evidenced by the lower levels of lipid peroxidation markers in this group of pigs. The literature, unfortunately, lacks data on the fatty acid composition of pork from pigs fed with thyme or thyme oil. Chickens receiving an aqueous extract of thyme (50 and 100 mg/kg of feed) and thyme powder (150 and 250 mg/kg of feed) had reduced levels of some saturated fatty acids in the breast muscle, accompanied by an increase in health-promoting unsaturated fatty acids [55]. Results obtained in pigs receiving extracts from other plants, such as rosemary (1 g/kg), indicate an increase in PUFA content in the longissimus lumborum muscle, especially linoleic, arachidonic, and docosahexaenoic acids [56].

## 5. Conclusions

The presence of thyme herb in diets with extruded flaxseed has a beneficial effect by influencing the antioxidant status of the blood and muscle and improving the lipid profile of tissues.

The study showed that a 3% inclusion of thyme in the diet significantly slows down oxidant processes, which may be a successful means of protecting meat against spoilage during storage. Furthermore, the 3% inclusion of thyme herb had a clear beneficial effect on lipid metabolism, so the levels of cholesterol and HDL cholesterol were reduced in both the blood and the longissimus dorsi muscle. In addition, the level of n-3 PUFAs in the muscles increased while the level of SFAs significantly decreased. The results of this study must be confirmed by further research.

## Figures and Tables

**Table 1:** Composition (g/kg) and nutritive value of growing and finishing pig diets [14].

Item	Grower			Finisher		
	C	T1	T3	C	T1	T3
Wheat	230	230	230	100	100	100
Rapeseed meal	80	80	80	120	122	125
Soybean meal	100	101	103	0	0	0
Thyme herb	0	10	30	0	10	30
Barley	483	472	450	647	635	612
Extruded flaxseed	40	40	40	40	40	40
Extruded soybean	20	20	20	20	20	20
Soya oil	20	20	20	0	0	0
Wheat bran	0	0	0	50	50	50
Dicalcium phosphate	3	3	3	0	0	0
Limestone	3	3	3	6	6	6
L-lysine chloride	1	1	1	2	2	2
Mineral–vitamin premix ^(1)^	20	20	20	15	15	15
Total	1000	1000	1000	1000	1000	1000
Analysed (g/kg):						
Dry matter	893.5	893.6	893.6	893.8	893.7	893.9
Crude protein	169.4	169.3	169.2	150.2	150.1	150.1
Ether extract	56.1	56.1	56.1	38.2	38.1	38.3
Crude fibre	49.3	49.4	49.6	57.4	57.5	57.7
Total lysine	10.3	10.3	10.3	9.06	9.06	9.06
Methionine + cysteine	6.52	6.52	6.52	5.89	5.89	5.89
Total phosphorus	5.57	5.57	5.58	5.17	5.17	5.17
Calcium	7.12	7.12	7.12	6.49	6.49	6.49
ME, MJ ^(2)^	13.07	13.07	13.06	12.56	12.56	12.55

^(1)^ 1 kg of mineral–vitamin premix contained vitamins: A 600,000 IU, D_3_ 60,000 IU, E 3000 mg, K_3_ 120 mg, B_1_ 120 mg, B_2_ 240 mg, B_6_ 240 mg, nicotinic acid 1600 mg, pantothenic acid 800 mg, folic acid 160 mg, biotin 10 mg, and B_12_ 1.6 mg; choline chloride 12 g, Mg 0.8 g, Fe 6 g, Zn 5.6 g, Mn 2.4 g, Cu 6.4 g, I 40 mg, Se 16 mg, Co 16 mg. ^(2)^ Metabolizable energy was calculated according to the equation proposed by Kirchgessner and Roth [15].

**Table 2:** Chemical composition of thyme herb (dry weight).

Item	Content
Nutrient g/100 g	
Dry matter	99.43 ± 0.54
Total protein	5.87 ± 0.143
Crude fat	3.92 ± 0.076
Crude ash	7.51 ± 0.109
Crude fibre	21.22 ± 1.65
Total polyphenols, mg GAE/g	12.32 ± 2.101
Total flavonoids, mg CE/g	1.50 ± 0.15
ABTS•+, mmol Trolox/g	2.11 ± 0.034
DPPH•, mmol Trolox/g	1.76 ± 0.066

DPPH• 2,2-diphenyl-1-picrylhydrazyl radical. ABTS•+ 2,2′-azinobis-(3-ethylbenzthiazolin-6-sulfonic acid) radical.

**Table 3:** Lipid profile parameters in the plasma of fattening pigs.

Item ^1^	Treatment	Feeding Groups ^2^			SEM ^3^	*p* _Value_		
		C	T1	T3		TRT ^4^	T ^5^	D ^6^
CHOL, mmol/L	Grower	2.10 ^a^	1.96 ^ab^	1.84 ^b^	0.031	0.003	0.066	0.093
	Finisher	2.50 ^a^	2.41 ^a^	2.05 ^b^	0.061	0.001	0.094	0.006
HDL-C, mmol/L	Grower	1.36 ^a^	1.27 ^b^	1.28 ^b^	0.015	0.046	0.049	0.955
	Finisher	1.58 ^a^	1.53 ^ab^	1.39 ^b^	0.029	0.012	0.074	0.013
%HDL	Grower	64.68	64.9	69.44	0.035	0.289	0.094	0.166
	Finisher	63.35	63.82	68.17	1.06	0.122	0.404	0.130
TG, mmol/L	Grower	0.681 ^a^	0.576 ^b^	0.633 ^ab^	0.033	0.015	0.074	0.062
	Finisher	0.686 ^ab^	0.575 ^b^	0.718 ^a^	0.011	0.039	0.088	0.013
LDL-C, mmol/L	Grower	0.433 ^a^	0.426 ^a^	0.272 ^b^	0.012	0.044	0.018	0.016
	Finisher	0.605 ^a^	0.615 ^a^	0.332 ^b^	0.044	0.003	0.234	0.005
CHOL/HDL	Grower	1.62	1.62	1.62	0.033	0.997	0.234	0.965
	Finisher	1.64	1.65	1.60	0.034	0.864	0.945	0.629

^a–b^ Means with the same superscript are statistically the same across all 3 treatments (*p* > 0.05) according to Tukey’s post hoc test. ^1^ CHOL–total cholesterol; HDL-C–high-density lipoprotein cholesterol; LDL-C–low-density lipoprotein cholesterol; TG–triacylglycerols. ^2^ There were 3 dietary treatments: a control diet (C) and diets T1 and T3, with 1% or 3% thyme, respectively. ^3^ SEM–standard error of the means. ^4^ TRT–*p*_value_ for overall effect of dietary treatment (diets C vs. T1 vs. T3). ^5^ T–*p*_value_ for thyme effect vs. control (diet C vs. T1 and T3). ^6^ D–*p*_value_ for thyme dose effect (T1 vs. T3).

**Table 4:** Biochemical parameters and enzyme activity in the plasma of fattening pigs.

Item ^1^	Treatment	Feeding Groups ^2^			SEM ^3^	*p*_Value_		
		C	T1	T3		TRT ^4^	T ^5^	D ^6^
TP, g/L	Grower	65.31	66.53	64.57	6.99	0.664	0.533	0.361
	Finisher	64.08	63.05	61.33	1.05	0.587	0.901	0.531
UA, mmol/L	Grower	0.077	0.073	0.082	0.003	0.555	0.927	0.200
	Finisher	0.067 ^b^	0.066 ^b^	0.088 ^a^	0.001	0.044	0.927	0.048
UREA, mmol/L	Grower	4.62	4.96	5.08	0.163	0.524	0.263	0.775
	Finisher	4.62 ^b^	4.53 ^b^	5.71 ^a^	0.190	0.009	0.263	0.011
CREAT, µmol/L	Grower	137.3	140.9	145.7	4.15	0.730	0.51	0.657
	Finisher	153.9	156.3	159.2	4.50	0.902	0.51	0.777
ALP, U/L	Grower	171.0 ^b^	184.6 ^ab^	204.8 ^a^	2.17	<0.001	0.045	0.039
	Finisher	127.3 ^b^	125.3 ^b^	139.2 ^a^	2.07	0.005	0.004	0.009
ALT, U/L	Grower	35.08	33.99	33.77	1.05	0.876	0.404	0.944
	Finisher	28.03	27.58	29.03	1.23	0.898	0.606	0.628
AST, U/L	Grower	33.89	32.31	28.03	1.14	0.187	0.099	0.133
	Finisher	24.26	23.87	29.96	1.16	0.054	0.127	0.060

^a–b^ Means with the same superscript are statistically the same across all 3 treatments (*p* > 0.05) according to Tukey’s post hoc test. ^1^ TP–total protein; UA–uric acid; UREA–urea; CREAT–creatinine; ALP-alkaline phosphatase, ALT-alanine aminotransferase; AST-aspartate aminotransferase. ^2^ There were 3 dietary treatments: a control diet (C) and diets T1 and T3, with 1% and 3% thyme, respectively. ^3^ SEM–standard error of the means ^4^ TRT–*p*_value_ for overall effect of dietary treatment (diets C vs. T1 vs. T3) ^5^ T–*p*_value_ for thyme effect vs. control (diet C vs. T1 and T3) ^6^ D–*p*_value_ for thyme dose effect (T1 vs. T3).

**Table 5:** Redox parameters in the plasma of fattening pigs.

Item ^1^	Treatment	Feeding Groups ^2^			SEM ^3^	*p* _Value_		
		C	T1	T3		TRT ^4^	T ^5^	D ^6^
SOD, U/mL	Grower	16.71 ^b^	16.38 ^b^	23.93 ^a^	0.957	<0.001	0.678	<0.001
	Finisher	35.67 ^b^	35.54 ^b^	37.98 ^a^	3.19	0.047	0.090	0.050
CAT, U/mL	Grower	4.71	4.61	5.06	0.014	0.360	0.090	0.214
	Finisher	5.06 ^b^	4.19 ^c^	6.19 ^a^	0.263	<0.001	0.678	<0.001
Vitamin C, mg/L	Grower	0.287 ^b^	0.293 ^b^	0.385 ^a^	0.316	0.001	0.042	0.005
	Finisher	0.224 ^b^	0.247 ^b^	0.293 ^a^	0.001	0.033	0.082	0.045
FRAP, µmol/L	Grower	12.38 ^a^	10.39 ^b^	12.62 ^a^	0.021	0.001	0.204	0.004
	Finisher	9.53 ^b^	13.37 ^a^	13.69 ^a^	0.338	0.038	0.004	0.222
LOOH, µmol/L	Grower	0.622	0.634	0.641	0.040	0.937	0.732	0.893
	Finisher	1.36 ^a^	1.04 ^ab^	1.19 ^b^	0.029	0.020	0.732	0.065
MDA, µmol/L	Grower	1.56 ^a^	1.59 ^a^	1.31 ^b^	0.012	0.001	0.204	0.004
	Finisher	1.36 ^a^	1.30 ^ab^	1.14 ^b^	0.033	0.009	0.204	0.058

^a–c^ Means with the same superscript are statistically the same across all 3 treatments (*p* > 0.05) according to Tukey’s post hoc test. ^1^ SOD–superoxide dismutase; CAT–catalase; FRAP–ferric reducing antioxidant power; LOOH–lipid peroxides; MDA–malondialdehyde. ^2^ There were 3 dietary treatments: a control diet (C) and diets T1 and T3, with 1% and 3% thyme, respectively. ^3^ SEM–standard error of the means. ^4^ TRT–*p*_value_ for overall effect of dietary treatment (diets C vs. T1 vs. T3). ^5^ T–*p*_value_ for thyme effect vs. control (diet C vs. T1 and T3). ^6^ D–*p*_value_ for thyme dose effect (T1 vs. T3).

**Table 6:** Redox parameters in the longissimus dorsi muscle.

Item ^1^	Feeding Groups ^2^			SEM ^3^	*p* _Value_		
	C	T1	T3		TRT ^4^	T ^5^	D ^6^
SOD, U/g	237.4 ^b^	244.8 ^ab^	267.6 ^a^	2.39	0.016	0.158	0.178
CAT, U/g	25.67 ^b^	26.18 ^b^	32.35 ^a^	2.4	0.039	0.507	0.026
Vitamin C, mg/g	0.252	0.257	0.251	0.01	0.711	0.825	0.817
LOOH, µmol/mg	3.16 ^a^	2.65 ^b^	2.10 ^c^	0.146	<0.001	0.031	0.041
MDA, nmol/mg	0.039 ^a^	0.032 ^b^	0.025 ^c^	0.003	0.038	0.115	0.049

^a–c^ Means with the same superscript are statistically the same across all 3 treatments (*p* > 0.05) according to Tukey’s post hoc test. ^1^ SOD–superoxide dismutase; CAT–catalase; LOOH–peroxides; MDA–malondialdehyde ^2^ There were 3 dietary treatments: a control diet (C) and diets T1 and T3, with 1% and 3 % thyme, respectively. ^3^ SEM–standard error of the means ^4^ TRT–*p*_value_ for overall effect of dietary treatment (diets C vs. T1 vs. T3) ^5^ T–*p*_value_ for thyme effect vs. control (diet C vs. T1 and T3). ^6^ D–*p*_value_ for thyme dose effect (T1 vs. T3).

**Table 7:** Fatty acid profile of total lipids in the longissimus dorsi muscle.

Item ^1^	Feeding Groups ^2^			SEM ^3^	*p* _Value_		
	C	T1	T3		TRT ^4^	T ^5^	D ^6^
Individual SFA (g/100 g of TFA)							
14:00	3.11 ^a^	2.67 ^b^	2.69 ^b^	0.090	0.044	0.016	0.825
16:00	22.62	22.74	22.65	0.088	0.878	0.714	0.768
18:00	12.79 ^a^	12.51 ^a^	11.43 ^b^	0.090	0.050	0.098	0.046
20:00	0.375	0.341	0.346	0.012	0.485	0.221	0.841
Individual MUFA (g/100 g of TFA)							
16:1 n-9	3.60	3.57	3.64	0.020	0.343	0.992	0.166
18:1 n-9	43.22	43.53	43.17	0.068	0.052	0.405	0.071
18:1 n-7	4.68	4.98	4.89	0.086	0.369	0.168	0.571
20:1 n-9	0.047 ^b^	0.040 ^b^	0.177 ^a^	0.013	0.049	0.423	0.011
Individual PUFA (g/100 g of TFA)							
18:2 n-6	4.87 ^b^	4.87 ^b^	5.09 ^a^	0.041	0.023	0.222	0.022
22:2	0.301	0.300	0.311	0.004	0.514	0.635	0.392
18:3 n-3	0.846 ^c^	0.978 ^b^	1.51 ^a^	0.014	0.039	0.024	0.048
20:3 n-3	0.782 ^b^	0.735 ^b^	1.34 ^a^	0.007	0.045	0.607	0.022
20:4 n-6	0.411	0.403	0.368	0.016	0.574	0.498	0.136
Other	2.34	2.33	2.26	0.156	0.977	0.907	0.875
SFA	38.88 ^a^	38.25 ^a^	37.11 ^b^	0.187	0.021	0.072	0.041
MUFA	51.56	52.12	51.88	0.125	0.189	0.093	0.331
PUFA	6.43 ^b^	6.55 ^ab^	7.24 ^a^	0.042	0.029	0.051	0.137
n-3	1.62 ^b^	1.71 ^b^	2.85 ^a^	0.023	0.028	0.275	0.027
n-6	5.29	5.28	5.46	0.045	0.194	0.409	0.056
n-3/n-6	0.310 ^b^	0.325 ^b^	0.523 ^a^	0.016	0.046	0.359	0.037
AL	0.600	0.565	0.555	0.007	0.059	0.015	0.813
TI	1.15 ^a^	1.10 ^ab^	0.975 ^b^	0.013	0.043	0.072	0.125
CHOL	111.8 ^a^	102.3 ^b^	99.25 ^b^	2.05	0.013	0.004	0.338

^a–c^ Means with the same superscript are statistically the same across all 3 treatments (*p* > 0.05) according to Tukey’s post hoc test. ^1^ TFA–total fatty acids; SFA–saturated fatty acids; MUFA–monounsaturated fatty acids; PUFA–polyunsaturated fatty acids; AI–atherogenicity index; TI–thrombogenicity index. ^2^ There were 3 dietary treatments: a control diet (C) and diets T1 and T3, with 1% or 3% thyme, respectively. ^3^ SEM–standard error of the means. ^4^ TRT–*p*_value_ for overall effect of dietary treatment (diets C vs. T1 vs. T3). ^5^ T–*p*_value_ for thyme effect vs. control (diet C vs. T1 and T3). ^6^ D–*p*_value_ for thyme dose effect (T1 vs. T3).

## Data Availability

Not applicable.
